# Prognostic Health Management Using IR Thermography: The Case of a Digital Twin of a NiTi Endodontic File †

**DOI:** 10.3390/s23094296

**Published:** 2023-04-26

**Authors:** Filippo Ruffa, Mariacarla Lugarà, Gaetano Fulco, Damiano Alizzio, Fabio Lo Savio, Claudio De Capua

**Affiliations:** 1 DIIES—Department of Information Engineering, Infrastructure and Sustainable Energy, Mediterranea University of Reggio Calabria, 89122 Reggio Calabria, Italy; 2 DICEAM—Department of Enviromental and Civil Engineering, Materials and Energetics, Mediterranea University of Reggio Calabria, 89122 Reggio Calabria, Italy; 3 DICAR—Department of Civil Engineering and Architecture, University of Catania, 95123 Catania, Italy

**Keywords:** thermography, digital twin, prognostic and health management, endodontic file, temperature, NiTi

## Abstract

Prognostic and health management technologies are increasingly important in many fields where reducing maintenance costs is critical. Non-destructive testing techniques and the Internet of Things (IoT) can help create accurate, two-sided digital models of specific monitored objects, enabling predictive analysis and avoiding risky situations. This study focuses on a particular application: monitoring an endodontic file during operation to develop a strategy to prevent breakage. To this end, the authors propose an innovative, non-invasive technique for early fault detection based on digital twins and infrared thermography measurements. They developed a digital twin of a NiTi alloy endodontic file that receives measurement data from the real world and generates the expected thermal map of the object under working conditions. By comparing this virtual image with the real one acquired by an IR camera, the authors were able to identify an anomalous trend and avoid breakage. The technique was calibrated and validated using both a professional IR camera and an innovative low-cost IR scanner previously developed by the authors. By using both devices, they could identify a critical condition at least 11 s before the file broke.

## 1. Introduction

Thermal imaging, also known as the Infrared Thermography Technique, is a contactless measurement technique that relies on studying the emission of bodies in the electromagnetic spectrum and the correlation between thermal radiation and surface temperature [1,2]. In a prior study, the authors introduced an infrared scanner technique for monitoring the structure of buildings to detect thermal gradients, which could result from insulation defects and thermal dispersion within internal environments. This method employs a single thermopile sensor to obtain thermal images. The sensor, mounted on a PAN and TILT scanning system, mimics the behavior of an FPA (Focal Plane Array) matrix. The user can select both the scanning range and angle, which determine the resolution of the matrix [3]. Thermal imaging is a non-contact monitoring (NCM) technique that is increasingly utilized in various applications, including identifying incipient damage as a preventive measure [4,5,6].

These methods are often used in Non-Destructive Test (NDT) applications where prompt detection is essential [7]. Sudden changes in surface temperatures can indicate an object or device’s imperfections, structural flaws, or stress concentration near the material’s fatigue limits in specific circumstances [8].

In mechanical industrial systems it is very common to find different components subjected to cyclic or time-variable load histories [9], such as rolling bearings and different types of brakes that work specifically by friction forces. The stress concentrations resulting from the dynamics of these components can be detected through temperature measurements [10]. In the electronics and IT industry, thermal performance is also considered to verify the functional stability of components and materials under working conditions [11,12,13,14].

Thermography is widely used in automatic controls and in the evaluation of the structural performance of components in the automotive sector [15,16]. Additionally, thermal imaging is used in the electronics and electrical engineering industry to assess critical components of boards [17] or switch boards [18] and the strength of the constituent materials [19,20]. Additionally, thermography is useful in different biomedical applications such as monitoring the temperature of patients [4] and the performance of devices that come in contact with them [21,22]. This paper focuses on the latter and specifically investigates the mechanical characteristics of some instruments used in endodontic therapy, which are prone to breakage during use, as the authors have previously studied [23,24].

In endodontic therapy, nickel-titanium alloy (NiTi) rotary files are widely used in the root canal treatment stage. This is due to the superelasticity and shape memory characteristics of NiTi, which allow it to produce desirable tapered shapes while maintaining flexibility and strength when rotating within curved canals [25]. Therefore, the NiTi alloy is able to recover its original shape under a range of high deformations. However, NiTi rotary instruments have a high risk of fracture during clinical operation [26]. There are various factors that may contribute to file failure, but cyclic fatigue and torsional stress are considered the two primary causes [27,28,29].

This paper implements real-time temperature monitoring of an endodontic file mounted on a torsional testing machine able to estimate reproducibly its fatigue life up to the failure. The ultimate goal is to develop an innovative strategy to predict and avoid file breakage. Indeed, starting from the above, the aim of the present work is to develop a new smart digital model for the NiTi alloy to implement a prognostic and health management system (PHM), driven by a digital twin (DT) approach. PHM aims to avoid failures by monitoring equipment conditions, analyzing data, and providing safety rules to be respected [30].

The results pertaining to Prognostic Health Management (PHM) are primarily observed in the manufacturing, automotive, energy, and aerospace industries. The primary impetus for developing new technologies is provided by the manufacturing and materials sectors [31].

Several approaches have been proposed to predict the behavior of objects, including the development of semantic models [32,33]. There are numerous studies in the literature focused on predicting and preventing target failure [34,35,36,37,38]. A similar case is presented in [39], where a predictive method based on fatigue tests is developed to enable control over the target. Recently, monitoring and diagnostic procedures have shifted towards technologies that incorporate the development of digital twins (DTs) [40,41].

In recent years, the demand for technological models that integrate the physical and digital space and guarantee seamless interaction between them has grown with the diffusion of the most recent information technologies [42,43,44,45,46,47,48,49,50,51]. The initial requirement was for a digital model that could interact with humans, even if only to receive input data. With the advent of IoT devices, which integrate sensors and software and are capable of connecting and exchanging data with other devices and systems via the Internet, it became possible to develop digital shadows, where the real world could be brought into the digital one autonomously. The concept of DT describes this integration of a physical system, virtual representation of the physical system, and technologies that allow data exchange between them in real-time, representing a revolutionary approach to decision making in many processes and the latest evolution of model simulation [52].

In this case, process-linked virtual copies can mirror physical systems and drive decisions more efficiently and optimally. The purpose of using DTs in PHM techniques is to provide accurate information to PHM, resulting from the fusion of data from the real world and virtual space. However, this is only possible if there is a bidirectional interconnection between the object and its virtual copy. Of course, the digital copy must reproduce the object with a high level of detail.

This work describes an innovative and non-invasive technique for early fault detection based on the use of digital twins and infrared thermography measurements. The technique is tested by implementing a monitoring of a NiTi endodontic file under controlled torque test to develop a strategy capable of avoiding breakage. The proposed DT is the virtual copy of a NiTi alloy endodontic file that, using experimental data measured from the real world such as environmental conditions and rotation speed, can predict the expected thermal behavior and create a reference thermal image of the real object with a suitable resolution. This image is compared with the real one acquired by an infrared temperature measurement system, and the developed algorithm is able to isolate high-risk working situations and intervene in the real world to avoid breakages.

The proposed technique has been tested and validated using two measurement devices: a professional FLIR SC325 taken as a reference and an improved version of an IR scanner previously developed by the authors in [3].

The paper is structured as follows: Section 2 describes in detail how the measurements in physical space are implemented and the setup used to make the IR measurements, Section 3 describes the architecture of the virtual space and discusses the implementation of the proposed digital twin, and Section 4 presents and discusses the obtained results.

## 2. IR Measurement in Real Space

The experimental case study presented in this paper involves the structural monitoring of a commercial endodontic file model (COLTENE CM 40.04) under torsion in a climatically controlled environment. These files are made of a nickel-titanium (NiTi) alloy, which undergoes crystalline phase changes from austenitic to martensitic based on applied mechanical or thermal loads [21].

**Figure 1:**
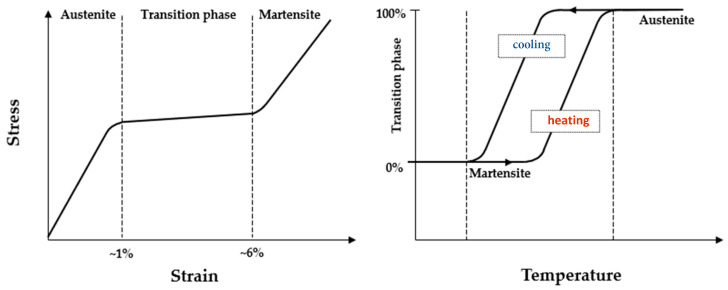
Material stress–strain and temperature correlation with crystalline phase transition for NiTi alloys.

As shown in Figure 1, which illustrates the crystallographic behavior of the alloy, the austenite/martensite transition range has lower stiffness and higher deformation capacity. A longer transition interval in comparison to the other two phases increases the alloy’s deformability, allowing it to operate flexibly within the curved root canals of teeth. Since mechanical loads trigger this transition, thermal loads can also induce it. The alloy’s superelasticity and shape-memory characteristics enable it to maintain flexibility and resistance during rotation within curved dental canals. The stress behavior during drilling operations is greatly influenced by the geometrical shape of these types of mechanical devices.

The proposed methodology for identifying potential fault situations is based on remote object temperature monitoring using infrared temperature sensors or a thermal imaging camera and coherence analysis between the thermal map predicted by the digital twin and the real-time measurement. All data processing operations are performed in the digital twin, as explained in Section 3. The digital twin uses all inputs from the monitoring system and updates the digital model of the object, producing an expected state that depends on both the input parameters and the history of the object. The predicted heat map of the object, calculated in the digital twin, is then compared with the heat map captured with the thermal imaging camera. The process is divided into two phases: the first analyzes the scene acquired in low resolution, and if there is a discrepancy in a certain area between the measurement and the model, it is possible to isolate the area and perform a high-resolution analysis in a second phase. The algorithm implemented in the digital twin, which triggers the low- and high-resolution analysis, is explained in detail in Section 3.2.

Thermographic measurements were performed both in a FLIR IR research environment using a FLIR SC325, taken as a reference instrument, and with an improved version of an IR scanner previously developed by the authors [3], described in the next section.

### 2.1. Experimental Setup

**Figure 2:**
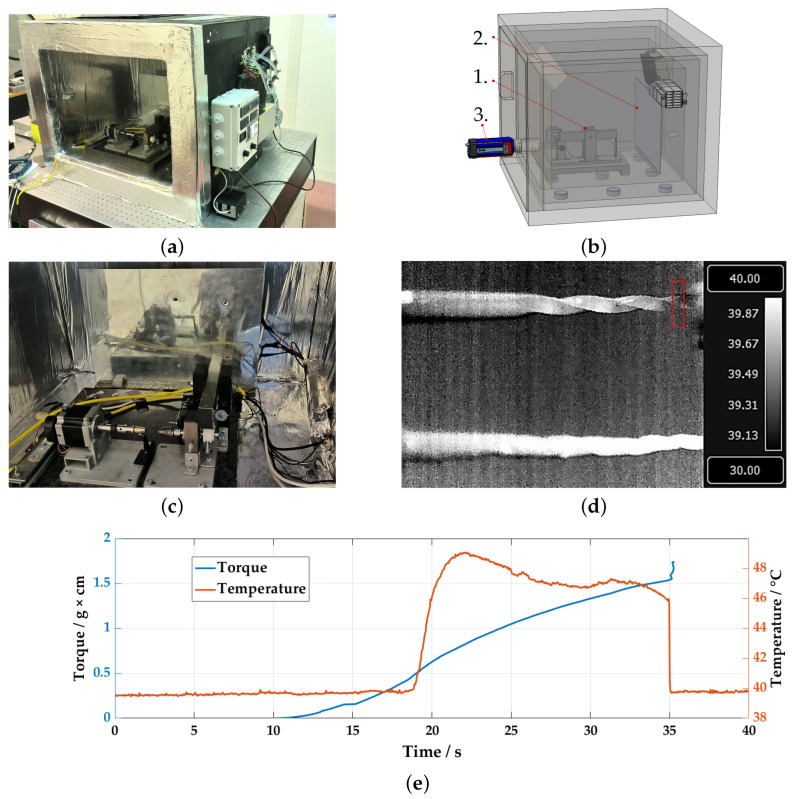
Experimental setup: (**a**) climatic chamber, (**b**) thermal measurement setup scheme (1. torquemeter, 2. radiant element, 3. thermal imaging camera), (**c**) torquemeter, (**d**) thermal frame, (**e**) torque and local temperature measurements on a 37 °C temperature-treated specimen.

Physical tests were conducted using a torque transducer specifically designed for testing endodontic files. Figure 2 illustrates the experimental setup used for measuring the breakage temperature of endodontic files under torque. The setup consists of an experimental torque transducer for endodontic files that operates in a controlled environment as shown in Figure 2a. The tests were monitored using thermal imaging (Figure 2b). The torque transducer was designed and manufactured in compliance with ISO 3630-1 standards, which require the use of a low-speed motor (2 rpm) and the connection of the endodontic file between two mandrels. One of these mandrels must have hardened steel jaws (to insert the file handle), and the other must be made of brass (to fix the tip of the tool at a length that can vary from 3 to 5 mm). The motor is driven by a 1063-Phidget Stepper Bipolar1-Motor board.

An incremental rotary encoder (HEDS-5500#A06) with quadrature output was mounted on the motor shaft to measure the angle of rotation relative to the torque required to generate file breakage.

To investigate the relationship between the mechanical properties of the NiTi alloy in endodontic files and the environmental conditions, a climatic chamber was designed and constructed. The chamber was created using SOLIDWORKS 2020 3D CAD and was simulated using Computational Fluid Dynamics (CFD). The chamber consists of a double envelope: the exterior is composed of XPS300 extruded polystyrene panels (40 mm thick), while the interior is made up of expanded polystyrene panels sintered with EPS-GRAF graphite (thickness 40 mm) and internally lined with aluminum foil (thickness 0.1 mm). A layer of rock wool (thickness 20 mm) is placed in the cavity between the two casings. The front panel also consists of a double layer (QQS300 + EPS-GRAF) and has a removable porthole in which the thermal camera lens can be inserted, specifically positioned to capture the position occupied by the torque meter spindles.

Inside the chamber, an aluminum radiating element is placed close to the rear panel and parallel to it. This element consists of an aluminum sheet, 0.2 mm thick, with an area of 50 × 30 mm^2^. A vertical septum made of the same material, placed at the back, separates a thermal area from a hygrometric one. In each of the areas, a heating resistor (DBK-HP06) with a total power of 100 W is fixed, each regulated by a thyristor SCR. The inlet of a 6-Peltier cell cooler (TEC1-12706) is located in the thermal zone, while the inlet of a humidifier is located in the hygrometric zone. An internal recirculation fan is placed in the upper center on the rear panel of the chamber and a water heat exchanger (Corsair Hydro Xd5-Rgb) is placed externally to dispose of the heat extracted from the cooler fan. The self-regulation system consists of a thermostat (STC1000) and a thermo-hygrometer (SHT2000), which regulate the temperature and humidity, respectively, by means of a set of sensors.

Figure 2c shows the experimental apparatus for the torque measurements, while Figure 2d displays the thermal imaging frame on the specimen. The latter image compares the thermal images of an endodontic file subjected to torsion load (top) and an unloaded file, or dummy (bottom). The acquisition reports the frame of a tested endodontic file on the upper side compared to a dummy file placed at rest on the lower side. The red rectangular box outlines the region of interest (ROI) where the torsion breaking of the endodontic file occurs. Data acquisition was carried out using NI-PXI and processed in the LabView environment (Figure 2e).

Several sets of tests were conducted on Coltene CM 40.04 specimens at both 37 °C (the temperature inside the mouth) and at 20 °C (room temperature). To minimize the error caused by metallic reflections, the files were treated with a matte black spray paint. As shown in the torque plot in Figure 2e, the endodontic file undergoes an initial elastic phase without any heat emission, followed by a change in convexity to the austenite–martensite transition phase. This crystallographic mechanism is indicated by the rapid increase in local temperature.

Thermographic measurements were performed in the FLIR IR research environment using a FLIR SC325 thermal imaging camera with a maximum rated noise equivalent temperature difference (NETD) of 50 mK. The frame size is 320 × 240 pixels, and a 16 × 12 mm field of view (FOV) was created using a 2× macro lens with a focal distance of 33 mm, resulting in a spatial resolution of 0.05 mm/px. Calibration was carried out based on the internal temperature of the climatic chamber once the temperature on the file had been reached and stabilized. Thermal recordings were made at an acquisition rate of 30 fps.

### 2.2. An Infrared Scanner Implementation

Another solution that was investigated for acquiring thermal images involves an improved version of a low-cost infrared scanner technique that was previously proposed by the authors in another work [3]. This implementation of the IR scanner involves two steps. In the first step, a static *m* × *n* FPA matrix of IR sensors acquires the thermal image of the object at a certain rate, determined by the user. The acquired image is then sent to the digital twin where it is computed and compared with the expected image. At this stage, it is not necessary to have a high resolution of the image. Therefore, the size of the FPA matrix can be minimized to save costs and processing time. If the algorithm detects a discrepancy between the real and the expected thermal map in one or more pixels, a higher-resolution scan is performed, only on the physical area to which the pixels in question refer. The high-resolution image thus obtained of the single frames is again compared with the reference in the digital twin.

**Figure 3:**
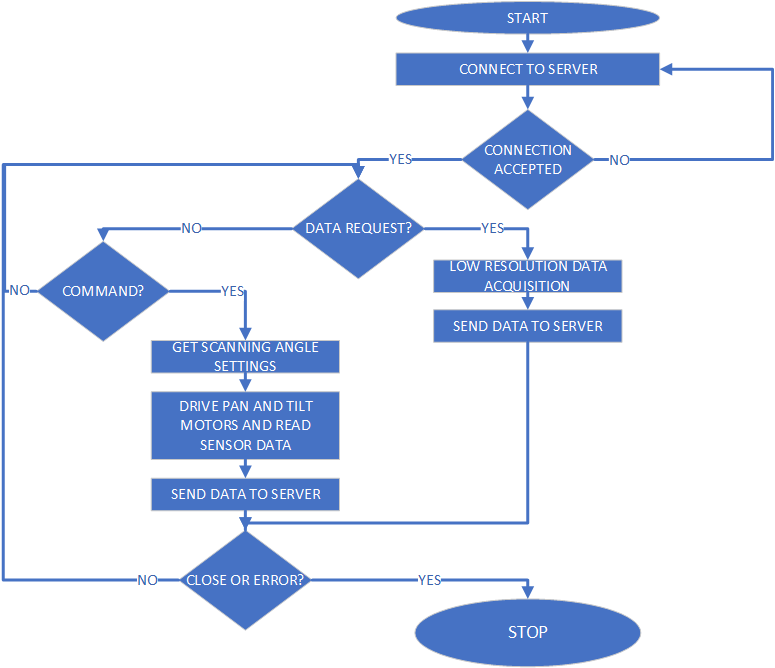
IR scanner flowchart.

The proposed solution for this high-resolution scan involves simulating an FPA architecture using a single thermopile temperature sensor mounted on a Pan and Tilt scanning system driven by two servomotors. The movement takes place along automatically set angles (both horizontally and vertically), detecting the temperature of the target points and simulating the behavior of an array of sensors (FPA architecture). Of course, such a system is much slower than a normal thermal imaging camera, as the higher the desired resolution, the higher the total number of readings that the sensor will have to carry out. However, the configuration flexibility makes it suitable for those applications that require adjusting resolution and measurement frames dynamically. The process described is managed by a microcontroller, which transmits the measurements taken to the remote server for processing and generates the necessary PWM signals to drive the two servomotors. The flowchart of the algorithm that controls the IR scanner is shown in Figure 3.

**Figure 4:**
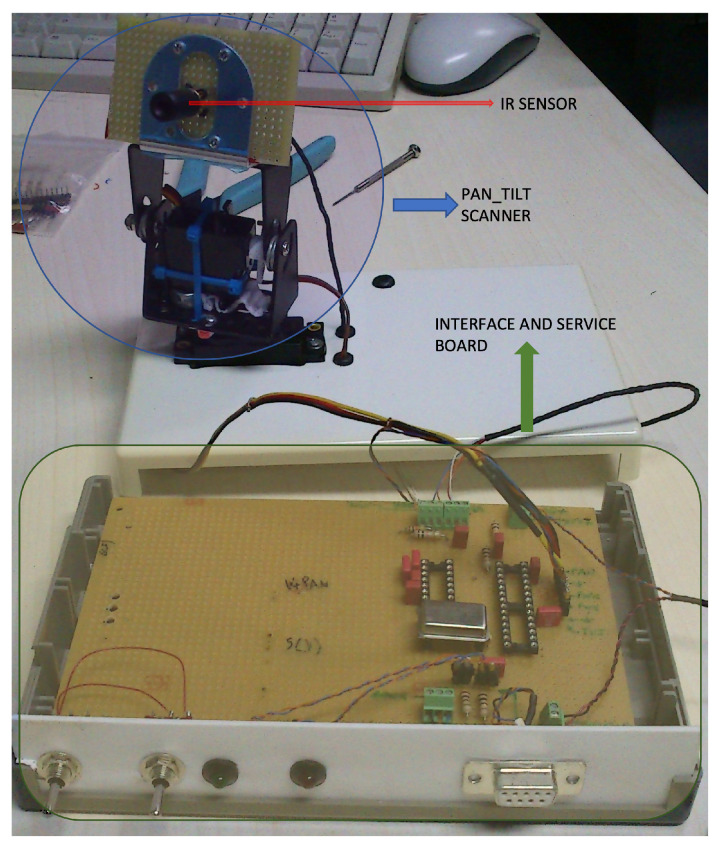
A first prototype of the proposed IR scanner.

A first prototype of the described system, as shown in Figure 4, has been developed and characterized, and its technical specifications for both the low- and high-resolution parts are presented in Table 1. The low-resolution part is performed using a static FPA matrix of IR sensors, while the high-resolution part uses a single thermopile temperature sensor mounted on a Pan and Tilt scanning system driven by two servomotors, simulating an array of sensors. The specifications provide details about the frame rate, spatial resolution, and temperature measurement range for both the low- and high-resolution parts of the system.

The specifications of the measurement uncertainty given in Table 1 are valid only under the condition that the temperature of the sensors is uniform. It is essential to keep in mind that the uncertainty data are derived from the thermal balance of the sensor, where all parts, including the casing, are at the same temperature without gradients. Therefore, it is essential to ensure that hot components or parts that can alter the thermal balance are not placed near the sensors during measurements. This effect is more pronounced for sensors with a small field of view, as they receive less energy from the object. The sensors used for implementing the IR scanner compensate for the internal thermal gradients, making them less sensitive but not immune to this issue. Therefore, it is crucial to design the measurement setup with care.

#### Limits of the System and Spatial Resolution

**Figure 5:**
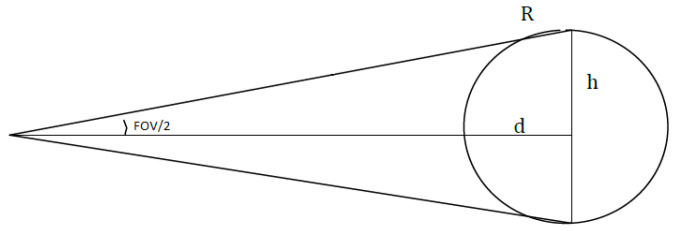
Geometry of the spot.

As mentioned, the measurement is carried out using a thermopile sensor, which returns the average temperature of the target area. We use the term “area” because each sensor has a certain beam opening, called the field of view (FOV). At a certain distance, the measured temperature is not related to a point, but to a spot whose diameter is directly proportional to the sensor’s FOV and the distance from the target. This places limits on the maximum achievable resolution. According to Figure 5, the spot diameter *d* is given by

(1) $$h=d\tan\left(\frac{\mathrm{FOV}}{2}\right)$$

(2) $$S=\pi h^{2}=\pi {\left(d\tan(\mathrm{FOV}/2)\right)}^{2}$$

where *h* is the radius of the measurement spot and *S* is its surface area. These equations are functions of the distance *d* and the FOV of the sensor. Therefore, to determine the minimum distance $\Delta X_{min}$ between two points on the wall in order to avoid information overlapping between adjacent readings, one can use Equations (1) and (2):

(3) $$\Delta x_{min}=2h=2d\tan\mathrm{FOV}/2$$

**Figure 6:**
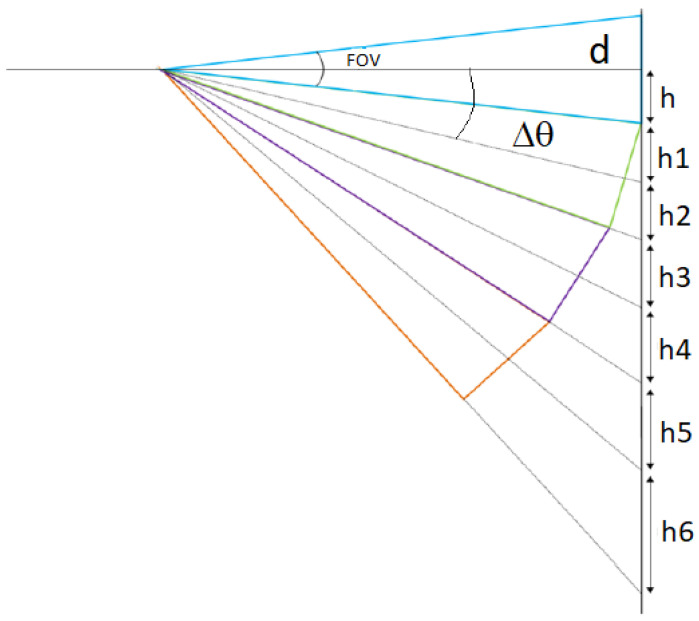
Spatial resolution of the system.

This means that the spatial resolution of the system is affected by the field of view of the sensor. Additionally, the spatial resolution worsens along the scanning angle due to the deformation of the reading spot. The maximum resolution is achieved when the sensor axis is perpendicular to the measured area. Figure 6 shows that the minimum displacement angle Δθ, which ensures that adjacent spots do not overlap, is equal to the FOV of the sensor.

(4) Δθ=FOV

while the diameter of the spot (*X*) grows as

(5) $$\Delta X_{n}=h_{2n-1}+h_{2n}$$

(6) $$h_{n}=d\left(\tan\left(\left(n+1\right)\mathrm{FOV}/2\right)-\tan n\mathrm{FOV}/2\right)$$

**Figure 7:**
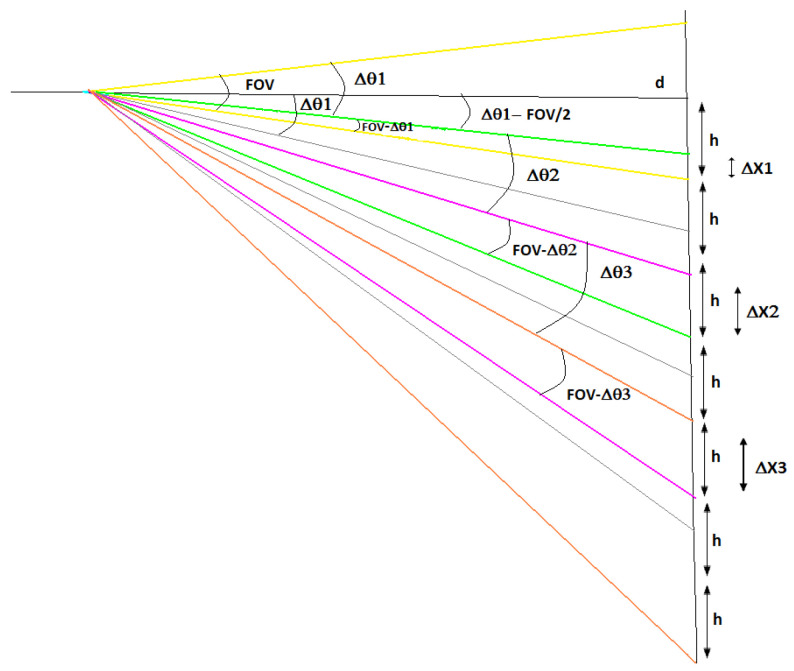
Spatial resolution of the system with the non-overlapping diameter of the spot fixed.

If the minimum step condition is not met, there will be some overlap of the respective target areas between two adjacent readings. While this improves the resolution, it also means acquiring redundant information since the measured temperature of each spot will contain information on the overlapping area. Several approaches to this problem are possible. One hypothesis is to fix the size of the non-overlapping diameter, as shown in Figure 7.

In this case, the diameter of the spot overlapped with the previous reading varies and increases along the scanning angle, as shown in Equation (7), and the displacement angle Δθ also varies. The angular position of the sensor at each step is calculated using Equation (8).

(7) $$\Delta X_{n}=d\left(\tan\left(\mathrm{FOV}/2+\sum_{i=1}^{n-1}\Delta \theta_{i}\right)-\tan\left[\left(-\mathrm{FOV}/2\right)+\sum_{i=1}^{n}\Delta \theta_{i}\right]\right)$$

(8) $$\theta_{n}=\arctan\left(\frac{nX}{d}\right)$$

The second approach involves fixing the percentage of information overlap between two consecutive readings. In this case, a constant percentage of the surface of the previous spot overlaps the surface of the next spot. The baseline case, with zero overlapping, is a special case of this approach. Therefore, the angle between two consecutive readings is fixed and can be calculated as follows:

(9) $$\Delta \theta =\frac{\left(100-\%overlap\right)}{100}\mathrm{FOV}$$

To further improve the resolution, modifying the sensor’s geometry can be an option. By physically limiting the sensor’s aperture through extending its chassis, the angle of incidence of incoming IR rays hitting the detector at the base of the chassis is reduced, effectively determining the new FOV of the sensor. An optical filter is then designed to filter out radiation that occurs with angles of incidence greater than the new FOV. However, introducing these extensions comes with physical limitations, as reducing the portion of incident radiation that reaches the detector affects its sensitivity. Below a certain optical power, the sensor may not be able to accurately detect the temperature. The sensor generates a voltage $V_{ir}$ at its terminals proportional to the intensity of the incident radiation, as shown in Equation (10) (where $T_{o}$ is the object temperature, $T_{a}$ is the ambient temperature, and *A* is a parameter that is a function of the body emissivity and the sensor’s FOV). At the same temperature, it generates a lower voltage (resulting in a lower temperature measurement) if the incident radiation is less intense.

(10) $$V_{ir}=A\left(T_{o}^{4}-T_{a}^{4}\right)$$

Therefore, if this strategy is adopted, the sensor must be re-characterized with the new optics to perform accurate temperature measurements. However, even though the system can be further improved, there is a lower limit to the FOV determined by the sensitivity of the sensor. Going below this limit (at least with the current technologies) is not possible.

## 3. Virtual Space

**Figure 8:**
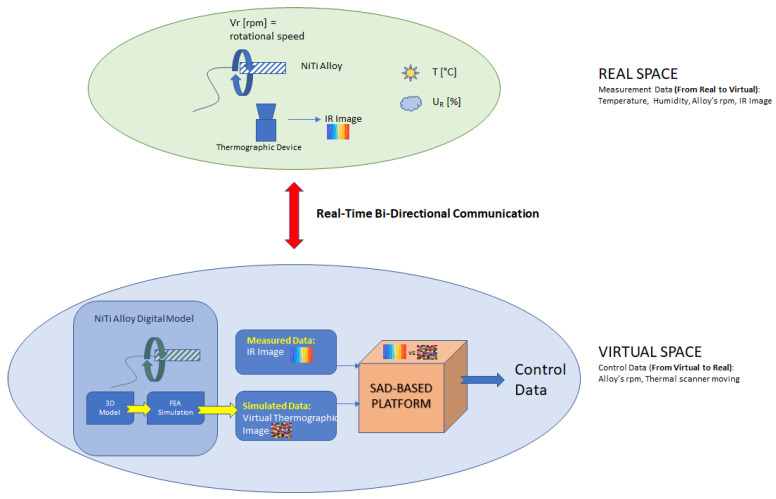
Real space and virtual space.

The smart control of the measurement system and the triggering of high-resolution acquisitions on specific target points is possible due to the continuous interaction between the physical space and the digital side. This interaction involves a two-way exchange of data between the virtual space, which implements the digital twin of the monitored object, and the real space. As shown in Figure 8, the digital twin is hosted on a remote server and connected to the real measurement instrumentation via the Internet network, communicating with it through the TCP/IP protocol. The digital twin receives monitoring data from the real space, updates the digital model of the object, and simulates it to produce an expected state that depends on the input parameters and past history of the object. The expected heat map of the object, computed in the digital twin, is then compared with that acquired with the thermal camera to isolate possible fault situations and intervene to avoid damage by turning off a smart plug that supplies power to the drill controlling file rotation. As shown in Figure 8, the digital twin of the NiTi endodontic file comprises its digital model and a PHM algorithm based on the computation of the Sum of Absolute Differences (SAD) metric.

### 3.1. Digital Model

**Figure 9:**
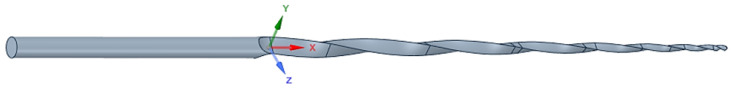
COLTENE 40.04 endodontic file 3D CAD model.

A 3D CAD model was designed, as shown in Figure 9, to simulate the torque behavior of the file using Finite Element Analysis (FEA). Mechanical ANSYS Parametric Design Language (APDL) was used to compute a hex-dominant mesh comprising 96,386 nodes and 23,050 elements, based on the cutter-shape CAD model of the endodontic file. The mesh was subjected to a torque static excitation using a fixed support 3 mm from the file tip and an imposed rotation of the stem section at 2 revolutions per second on the other side. The analysis settings were configured for non-linear quasi-static iterative evaluation over the entire length of the experimental tests. FEA was employed in the ANSYS environment to determine the fracture quantities in terms of stress concentration, heat emission, and superficial temperature of the NiTi alloy close to the fracture point. Additionally, a specific numerical model was established to predict deterministic fracture of the endodontic file, which was calibrated via statistical enhancement. The torque, torsion angle, and temperature at the fracture point were monitored to identify performance at different ambient temperatures and to update the digital model in real time.

**Figure 10:**
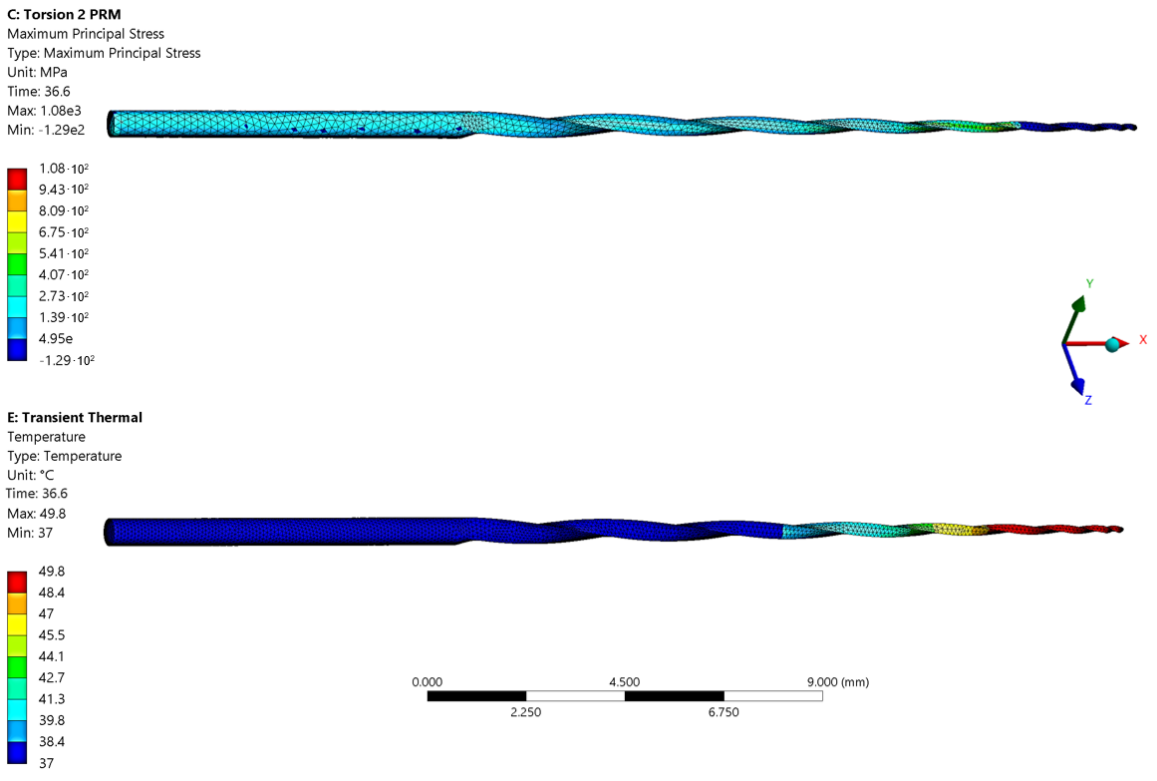
FEA results on COLTENE 40.04 endodontic file 3D CAD model.

**Figure 11:**
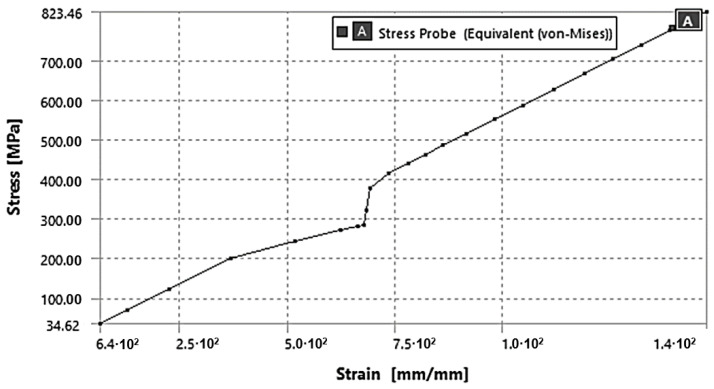
Numerical stress–strain curve relative to the endodontic file twisting fracture zone.

Figure 10 displays the results obtained from the numerical simulation, which was carried out to replicate a 2 rpm rotation for 36.6 s, as observed in the experimental tests. The upper graph illustrates the stress distribution on the undeformed model, and the lower graph shows the corresponding temperature increase. Figure 11 presents the stress–strain equivalent values attained by the surface of the numerical model near the fracture zone.

### 3.2. Fault Detection Algorithm

The process consists of two phases. In the first phase, the scene captured at low resolution is analyzed. If there is a discrepancy in a certain area between the measured and modeled data, the algorithm isolates the area and conducts a high-resolution analysis in a second step.

To elaborate, the algorithm considers a predetermined number of thermal image frames, allowing for the comparison of the average temperature of each frame with the corresponding frame in the model. Using the average temperature of a frame has a dual advantage; it mitigates the impact of measurement noise and reduces the computational effort required for comparison operations. In this case, the comparison is conducted between two averaged macro-pixels rather than two high-resolution matrices of pixels.

To measure the discrepancy between the actual and expected thermal behavior of the monitored object, the algorithm employs the Absolute Difference (AD) index. The comparison is made using the absolute differences method, as shown in Equation (11), where $R_{ij}$ represents the single averaged pixel of the measured thermal image and $M_{ij}$ is the averaged computed pixel for the same area in the model. If the value of the AD index exceeds a set threshold, which depends on the specific application, the algorithm detects a potential failure condition.

(11) $$AD_{ij}=\left|R_{ij}-M_{ij}\right|$$

**Figure 12:**
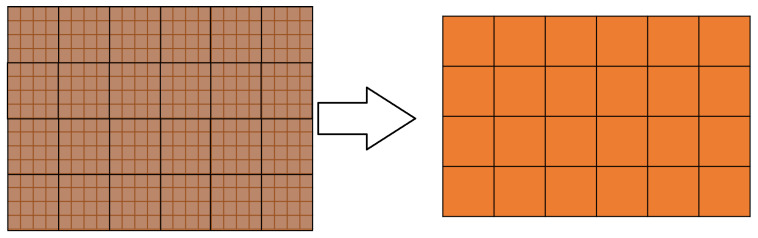
Computation of the averaged pixels.

To apply Equation (11) and calculate each element of the AD matrix, the original images must be processed. This involves dividing the images into n×m frames, where *n* and *m* are the number of rows and columns in the AD matrix, respectively. Each frame is then divided into w×h pixel windows, where *w* and *h* are the dimensions of the window. The average temperature is calculated for each window, and the resulting data are used to populate the corresponding element in the AD matrix. This procedure is illustrated in Figure 12. By using this method, a more accurate comparison between the measured and modeled data can be obtained, while reducing the computational effort required for the comparison operation.

The second phase is triggered if there is a significant mismatch in one or more frames and involves analyzing the discrepancy between the measured and expected thermal images at the highest possible resolution, in the selected frames. The Sum of Absolute Differences (SAD) method was chosen as the metric for discrepancy detection due to its simplicity and efficiency [53,54,55]. SAD evaluation is a widely used technique to analyze image coherence for various purposes such as object recognition, generation of disparity maps for stereo images, and motion estimation [56]. As shown in Equation (12), the SAD index measures the similarity between the thermal image measured with the IR camera and the image expected in the digital twin for the selected frame. If the two images are similar, the value of SAD(R,M) will be low, ideally zero. However, if there is a mismatch between the two images, the value of SAD(R,M) will be higher.

(12) $$SAD\left(R,M\right)=\sum_{i,j}\left|R_{ij}-M_{ij}\right|$$

**Figure 13:**
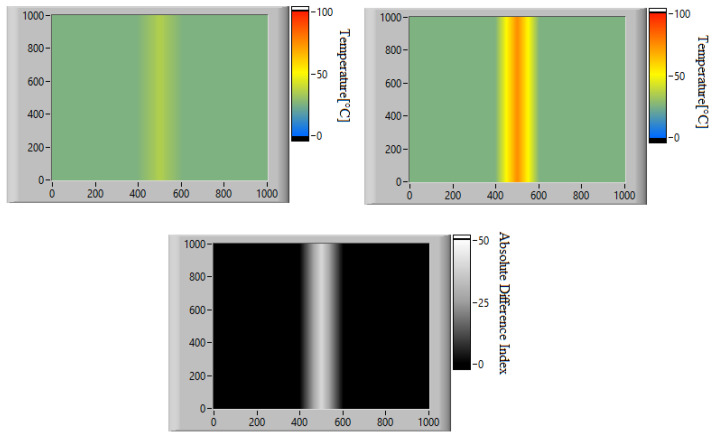
Exampleof two thermal images (top) and their Absolute Differences matrix (bottom)—Image dimension: 1000 × 1000 px.

If the value of SAD(R,M) is higher than a pre-set threshold, which depends on the specific application, it indicates that there is a significant discrepancy between the measured temperature and the expected temperature in one or more areas of the frame. Using this criterion, the algorithm determines whether the detected mismatch is indicative of a possible fault or not. In the case of a potential fault, the algorithm activates a smart plug to switch off the drilling system. Additionally, the algorithm calculates the disparity map (Figure 13) using Equation (11). This enables the identification and isolation of areas with a high probability of fault.

**Figure 14:**
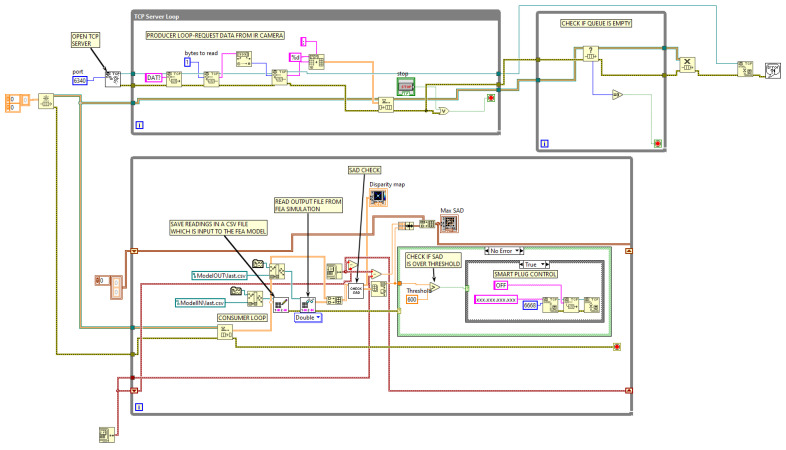
Blockdiagram of the implemented VI.

**Figure 15:**
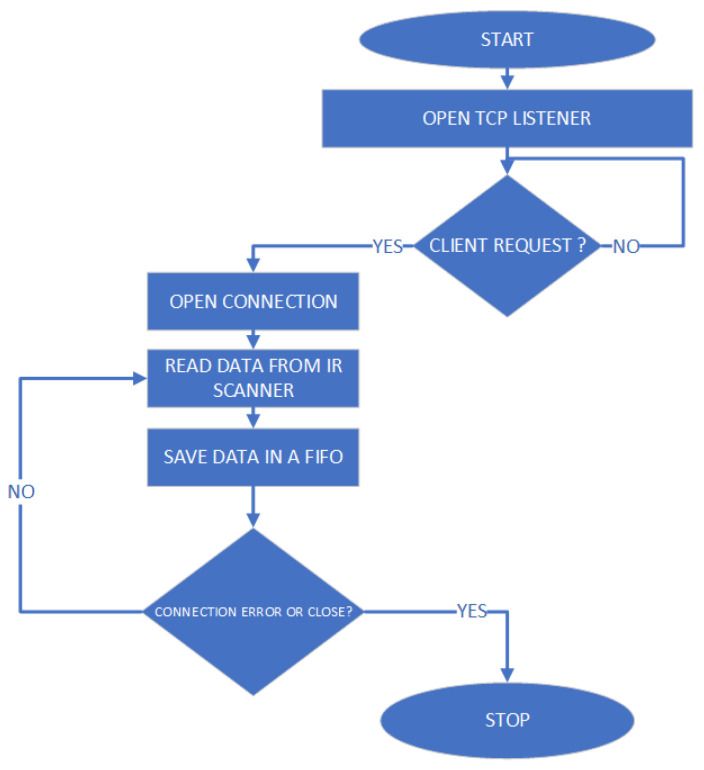
Digital twin communication flowchart.

**Figure 16:**
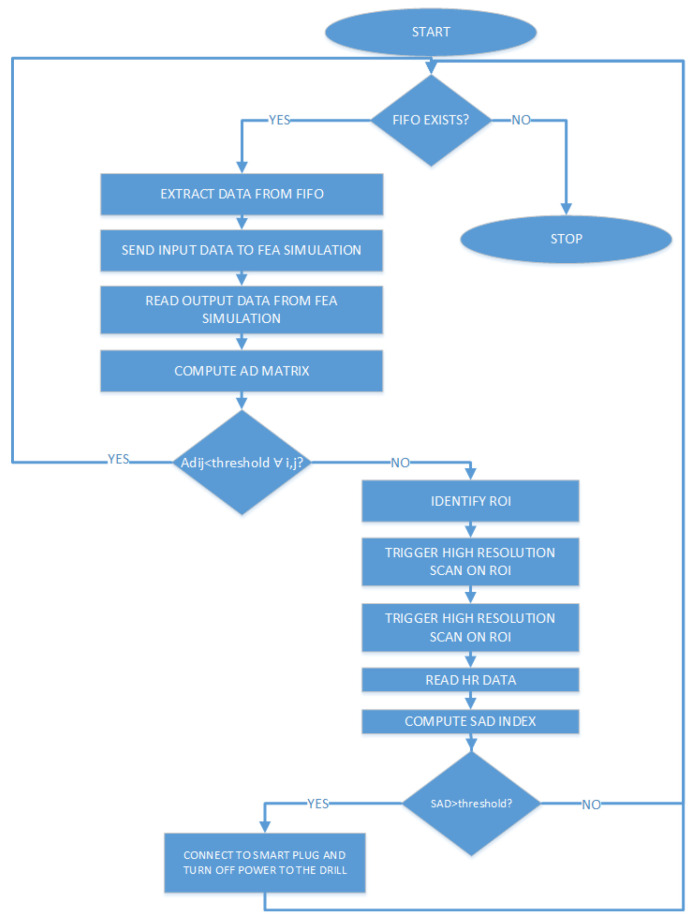
Digital twin data processing flowchart.

The authors implemented the proposed algorithm in NI LabVIEW, and the VI developed for this purpose is shown in Figure 14. This VI is responsible for managing data communication with the real space via the TCP/IP protocol, as well as data processing using the method described in the previous section. Real-time transfer of information to and from the physical model implemented in ANSYS is achieved using file streaming. To provide a better understanding of the algorithm, flowcharts for the communication and data processing parts are presented in Figure 15 and Figure 16, respectively.

## 4. Results

The technique presented in this paper aims to detect potential failure situations in structures and mechanical parts at an early stage by monitoring the object’s temperature and data processing in its digital twin. The technique, which is described in detail in Section 3 and Section 3.2, was calibrated and validated using the experimental setup described in Section 2.1. The setup involved monitoring a NiTi endodontic file under a controlled torque test.

The algorithm and behavior of the material were characterized by operating at two different ambient temperature set points, i.e., 20 °C and 37 °C, demonstrating similar performances in both cases.

### 4.1. Calibration of the Algorithm and Choice of the SAD Threshold in Post-Processing

**Figure 17:**
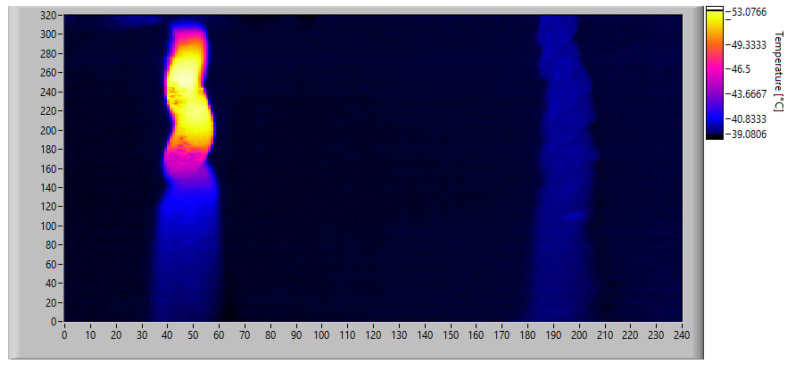
Actual thermal behavior of the endodontic file at the instant of maximum heating. Image resolution: 320 × 240 px.

**Figure 18:**
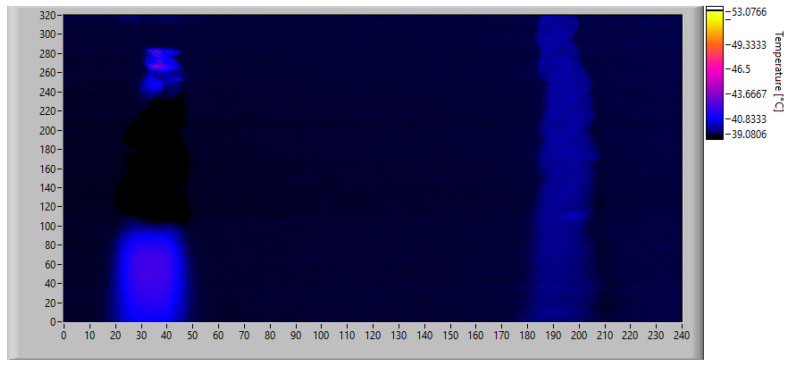
Actual thermal behavior of the endodontic file at the instant of breakage. Image resolution: 320 × 240 px.

To calibrate the algorithm, the data obtained from monitoring the unit under test at 37 °C were post-processed to analyze the performance of the proposed technique and to adjust the thresholds for the detection of potential failure situations. Figure 17 and Figure 18 show the actual thermal situation monitored with the thermal imaging camera at the instant of maximum heating and file breakage, respectively.

**Figure 19:**
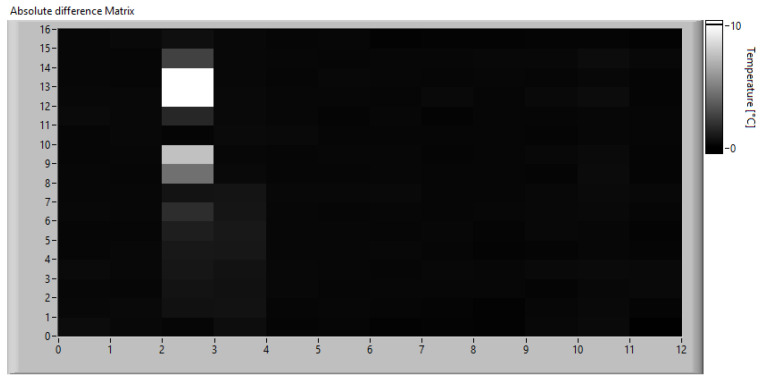
Absolute difference matrix at the instant of maximum heating.

Once the monitoring data were acquired, the algorithm was calibrated by simulating real-time operation and applying the algorithm described in Section 3.2. First, a low-resolution analysis was performed using fixed frames averaging 16 × 12 pixels. Considering that the original images have a size of 320 × 240 px, each computed frame is obtained by dividing the original images into 20 × 20 px windows and calculating the average temperature in each. This operation is performed on both the expected heat map obtained from the simulation of the physical model and on the real one acquired with the thermal imaging camera. The absolute difference matrix obtained using Equation (11) is shown in Figure 19.

**Figure 20:**
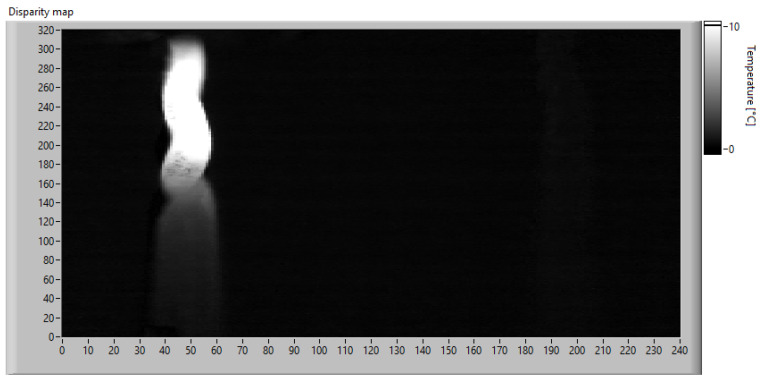
Disparity map at the instant of maximum heating.

**Figure 21:**
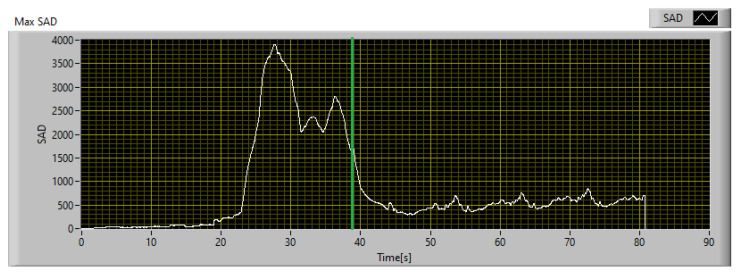
Maximum SAD trend during the acquisition.

Since the goal of this phase is to calibrate the SAD threshold for the specific application, the trend of SAD was observed throughout the acquisition time. The SAD index was calculated on the original images using 20 × 20 px windows and Equation (12). The disparity map computed at the instant of maximum heating is shown in Figure 20, while Figure 21 shows the maximum SAD trend, i.e., the highest value of all the computed SAD indices, throughout the entire acquisition time.

From Figure 21 it can be observed that the SAD value remains less than 100 under normal conditions and rapidly increases when the thermal behavior of the object deviates from the expected behavior. Once the SAD value reaches its maximum value, it remains on high values until the failure occurs. After the failure, the temperature and SAD value both decrease rapidly. The endodontic file breakage occurred 34 s after the start of monitoring and is identified by the green line in Figure 21. Considering that the SAD value increases rapidly when there is a discrepancy between the measurement and the model, and taking into account the relative uncertainty in both measurement and model forecasts, an SAD value of 600 can be considered as a reference threshold for the detection of a potential fault. In this specific case study, the chosen threshold is high enough to avoid false detections due to oscillations of the SAD value, but low enough to detect a potential fault 15.7 s before the actual failure of the endodontic file. This is sufficient to intervene and avoid damage to the component.

Taking the aforementioned situation as a reference, the possibility of using the described technique with a low-cost implementation was considered. The technical characteristics of this implementation are described in Table 1. To do so, an acquisition was simulated using the IR scanner described in Section 2.2, which is summarized in Table 1, on the same data (obtained in real tests with the reference thermal camera). Taking into account that the frames acquired by the thermal imaging camera, such as the one shown in Figure 17, cover an area of 16 × 12 mm^2^, the positioning of the IR scanner must be set to detect approximately the same area within its Field of View (FOV). In the simulations performed, the IR scanner was considered installed at a distance of 200 mm from the target, and the measuring area falling within the FOV was calculated using Equation (1). The sensor used for the low-resolution acquisition has an FOV of 57°, which means that at a distance of 200 mm, the measured spot has a diameter of 21.7 mm. The sensor is a 16 × 12 FPA matrix, and each element covers a portion of the measured spot corresponding to an area of 1.36 × 1.81 mm^2^.

**Figure 22:**
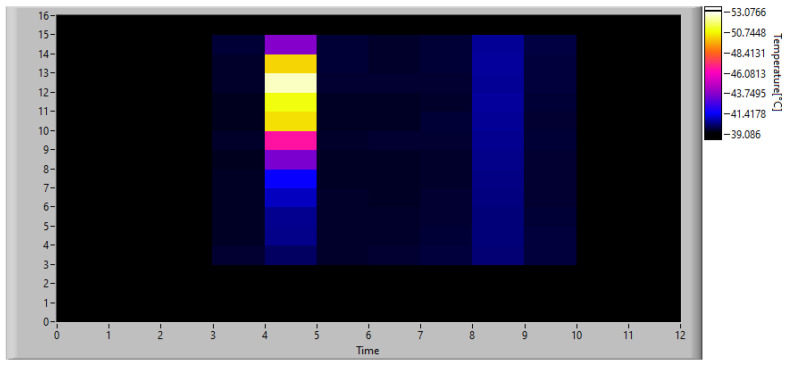
Thermal image of the endodontic file at the instant of maximum heating simulating an acquisition with the IR scanner at low resolution. Image resolution: 16 × 12 px.

**Figure 23:**
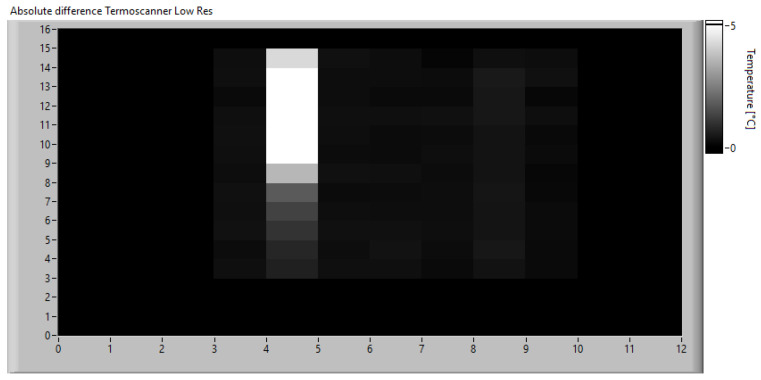
Absolute difference matrix at the instant of maximum heating simulating an acquisition with the IR scanner at low resolution.

Considering that the reference image has a resolution of 0.05 mm/px for each pixel measured by the simulated IR scanner, using the configuration described, it represents the average temperature of a 27 × 36 px window of the original image. Since the target area measured with the IR scanner is larger than that of the reference, the missing spaces of the latter have been filled by inserting dummy pixels. Using this configuration, an acquisition with the same time constraints as the real scanner was simulated, and Figure 22 shows the thermal image obtained at the instant of maximum heating, while Figure 23 shows the Absolute Differences matrix calculated at the same time.

The limit threshold used to trigger the high-resolution acquisition on the detected anomalies is set at 5 °C. Once this threshold is reached, the algorithm initiates a simulated IR scan with the 5° FOV sensor on the selected area. The target area, calculated using Equation (1), has a diameter of 1.7 mm, corresponding to a 34 × 34 px window in the reference image. At this stage, a scan on the target area is simulated using the solution with a fixed 90% information overlap, as described in Section 2.2. This implies that the angular step is fixed using Equation (9), resulting in a step of 0.5°. The non-overlapping distance is calculated using Equation (1) and is equal to 0.17 mm, while the overlapping spot portion varies along the scanning angle following Equation (7).

**Figure 24:**
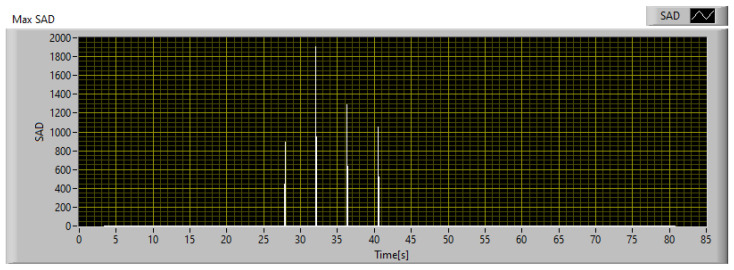
Maximum SAD trend during the acquisition with the IR scanner.

Considering that the anomaly detected in the low-resolution image corresponds to an area of 1.36 × 1.81 mm^2^, the described HR scanning setup requires a 6 × 9 scan matrix to cover the entire area of 1.36 × 1.81 mm^2^. The IR scanner is simulated by reproducing its expected behavior in the software, and the resulting performance is shown in Figure 24. As can be seen, using an SAD threshold of 100, the anomaly is detected 11 s before failure, which is sufficient to take corrective measures, while its maximum level is registered 6.5 s before failure.

**Figure 25:**
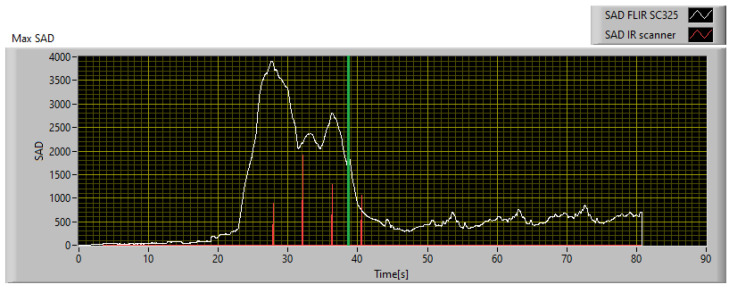
Comparison between the maximum SAD trend obtained with the thermal camera and the IR scanner.

To compare the performances of the implementation of the technique, obtained with the professional thermal imaging camera and the low-cost IR scanner, respectively, the two indices should be evaluated in the same conditions. In fact, the maximum SAD calculated on the reference data stream and shown in Figure 21 is computed using 20 × 20 px windows, while that of the IR scanner is computed on 6 × 9 px windows. To compare both under the same conditions, the SAD measured with the IR scanner must be multiplied by a factor 7.41. The result so obtained is shown in Figure 25. As can be seen, the IR scanner is slower and less sensitive in detecting anomalies, compared to the reference. In fact, using an SAD threshold of 600, which is the threshold defined for operations with the reference camera, the IR scanner detects the anomaly 4.7 s later. In any case, considering that the potential fault is detected 11 s before failure, it is still possible to intervene avoiding damage to the monitored part. Therefore, the proposed technique has been calibrated considering two different implementations, and its effectiveness has been demonstrated for both; the first guarantees better performances and greater responsiveness, while the second instead is a good compromise between performances and implementation costs.

### 4.2. Validation of the Algorithm in Real-Time Operations

**Figure 26:**
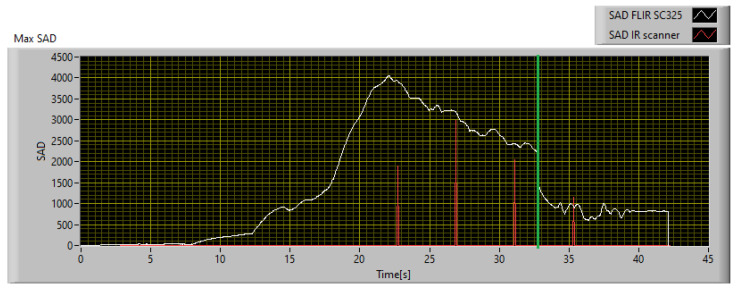
Comparison between the maximum SAD trend obtained with the thermal camera and the IR scanner in the validation test at 20 °C.

To demonstrate the functioning of the algorithm, whose SAD threshold was calibrated as mentioned in Section 4.1, operating at a different ambient temperature than that used for SAD threshold calibration, a similar endodontic file was monitored using the experimental setup described in Section 2.1 in a controlled environment with an ambient temperature of 20 °C. The file was monitored in real time using both the reference camera and the IR scanner described in Section 2.2 and implementing the digital twin described in Section 3. The obtained results and the comparison between the two implementations are shown in Figure 26. It can be seen that the file break occurred 31.7 s after the start of monitoring and is represented in Figure 26 with a vertical green line.

As demonstrated using the previously calibrated setup, the anomaly was detected 19.9 s before the actual break using the implementation with the professional thermal imaging camera, and 10.5 s before using the IR scanner, which confirms the results obtained during the calibration phase. The digital twin’s ability to prevent file breakage was demonstrated by implementing remote control of a smart plug and shutting off power to the drill when the SAD value crosses the set threshold. Using an SAD threshold of 600, the algorithm was able to avoid file breakage in all 20 validation tests performed. To better evaluate all possible issues related to the contribution of measurement uncertainty, the worst implementation in terms of performance was considered, i.e., the one that uses the IR scanner, and the data obtained from a single test were corrupted by injecting Gaussian white noise centered on the measured value with a standard deviation equal to the uncertainty declared in Table 1. The operation was repeated 1000 times to obtain a proper statistical sample, and the results are summarized in Table 2.

**Figure 27:**
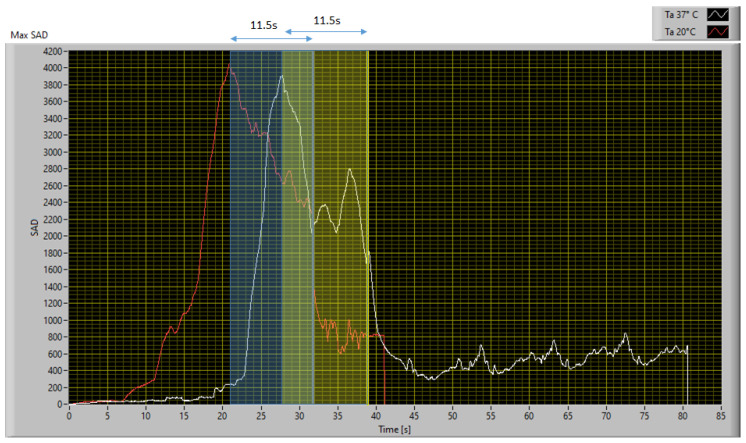
SAD trend obtained testing the file at an ambient temperature of 20 °C and 37 °C.

Furthermore, by repeating ten tests at each of the two different ambient temperature set points, it was possible to observe and characterize the behavior of the material. During the martensitic phase, which occurs during heating, the file is damaged and then breaks during the cooling phase, while returning to the austenitic phase. It was possible to experimentally determine a time window between the instant of maximum heating, identified by the peak in the SAD index trend, and the instant of rupture. As shown in Figure 27, in both situations, the file broke 11.5 s after the maximum heating.

### 4.3. Critical Analysis and Discussion

The proposed technique employs non-invasive infrared thermography to measure temperature distribution on a target object and a fast-computation metric, the SAD metric, to measure the discrepancy between the expected and actual heat map. The expected behavior is obtained in real time in the virtual space by running FEA simulations on the digital model, which is the core of the digital twin. The model is updated in real time using measurement data and produces an expected heat map of the object step by step, which is compared using the SAD metric with the one obtained from the thermal monitoring system. Another innovative aspect of this technique is that it can be implemented with low-cost measurement systems made with common consumer electronic components. The authors proposed an implementation consisting of an infrared scanner that uses a low-resolution thermopile sensor matrix for a rough estimate of the thermal distribution on the object and a single thermopile sensor mounted on two servomotors to simulate an FPA architecture for high-resolution scanning. This allows for obtaining, at a low cost, a flexible system that enables the user to dynamically set both the resolution and the frame size.

The technique was calibrated and validated by monitoring controlled torque testing of NiTi endodontic files in a climate-controlled environment. The SAD threshold was calibrated at a temperature set point of 37 °C. The algorithm was then validated at a different temperature set point to demonstrate that the SAD threshold of 600 defined for one ambient temperature set point is still valid for another. The ambient temperature chosen for algorithm validation was 20 °C. The algorithm was calibrated in post-processing on the results obtained at 37 °C, monitoring the entire test procedure using a FLIR SC325 thermal imaging camera. Using an SAD threshold of 600, the algorithm detected a fault 15.7 s before file breakage. The same criteria were used to calibrate the algorithm for a simulated version of the IR scanner proposed in Section 2.2, using the measurement dataset obtained with the professional IR camera. Using this configuration, even though the performance was worse and the fault detection occurred 4.7 s after the reference, it still occurred 11 s before the failure. The algorithm, thus calibrated, was validated by monitoring a similar endodontic file during a stress test at 20 °C, confirming the robustness of the technique to automatically detect a potential defect. In this case, the detection occurred 19.9 s and 10.5 s before the file was damaged for the reference camera and the implementation of the low-cost IR scanner, respectively. To consider all possible situations and the contribution of the measurement uncertainty, a statistical analysis was performed on the data obtained with the worst performing system, i.e., the IR scanner, by affecting the data with a Gaussian white noise centered on the measured value and with a standard deviation equal to the type B uncertainty associated with the sensors used. A total of 1000 simulations were performed to have a significant statistical sample, and the results confirmed that the measurement uncertainty did not affect the goodness of the results obtained.

Furthermore, it was possible to use the proposed technique to characterize the behavior of the material. By carrying out a series of 10 tests for each ambient temperature set point, it was possible to identify a constant time window that elapses between the instant of maximum heating, identified by the maximum peak in the trend of the SAD index, and the instant of rupture, which is 11.5 s.

## 5. Conclusions

The early detection of warning situations that may cause damage and the improvement of device reliability pose significant challenges for the future. In this paper, the authors propose an innovative predictive technique implemented at the digital twin level. This technique relies on real-time model–measurement interaction to detect unexpected thermal behaviors, which may be closely related to, or precede, structural failure.

The proposed technique focuses on a specific application: monitoring an endodontic file under torsion conditions to develop a strategy that can prevent its breakage. The authors developed a digital twin of a NiTi alloy file that receives measurement data from the real space and generates the expected heat map of the object under working operating conditions. In the real space, the endodontic file is monitored using the IR thermography technique. The acquired data are sent in real time to the digital twin, which compares its virtual image with the real one acquired by the IR camera. This approach enables the identification of an anomalous trend and helps to prevent breakages. With both devices, it was possible to identify a critical condition at least 11 s before the file broke, which was sufficient to intervene and avoid damage.

Moreover, the authors conducted a series of 10 tests for each ambient temperature set point to characterize the material behavior using the proposed technique. They observed that the instant of rupture, in all cases, occurred 11.5 s after the maximum peak in the trend of the SAD index. The use of an innovative predictive technique for Prognostic Health Management, which is based on the co-monitoring of the reality model, can help create a new generation of digital twins. These twins will increase their ability to interact and have an impact on reality in the smart operations panorama.

Although the proposed technique has been calibrated and validated in a specific case study, i.e., the monitoring of a NiTi endodontic file during a torque test under specific operating conditions, future perspectives will focus on extending the application of the proposed technique to the monitoring of other and more complex structures. Other future developments will concern a more in-depth characterization of the NiTi files. This will correlate specific thermal behavior with specific crystalline changes in the material, aiming at the optimization of the modeling phase. Finally, future work will focus on the optimization of the proposed algorithm through the introduction of other innovative metrics.

## Figures and Tables

**Table 1:** Technical specifications of the IR scanner prototype implemented.

	Low Resolution	High Resolution
Structure	Fixed	Mounted on Pan and Tilt Scanning system
Scanning angle [°]		30–150 both for X and Y
Minimum step [°]		0.3
n. of pixels per reading	16 × 12	1
FOV [°]	57	5
Min. refresh time [s]	0.016	0.06∗(Xspan∗Yspan)/FOV
Operating temperature range [°C]	−40/+125	−40/+125
Target temperature range [°C]	−40/+300	−40/+300
Temperature resolution [°C]	0.01	0.01
Type B uncertainty ^1^ [°C]	Max. ± 1	Max. ± 1

^1^ The stated uncertainty applies to a sensor operating in air within an ambient temperature range of 0 °C to 50 °C, and with a target temperature range of 0 °C to 100 °C.

**Table 2:** Results of the validation tests.

	Results
Endodontic file breaking time [s]	39.7
Average fault detection time [s]	24.17
Standard deviation of the detection time [s]	0.53
Average SAD level at detection [°C]	1883.76
Standard deviation of SAD level at detection [°C]	43.55

## Data Availability

Not applicable.

## References

[1] Longo M.J., Akerlof C., Allersma M., Becchetti F., Bucksbaum P., Crane R., Grafe A., Hendel F., Lee M., Levy K. (2005). Waves, Light and Heat Laboratory Manual for Physics.

[2] Liou K.N. (2005). An Introduction to Atmospheric Radiation.

[3] Ruffa F., Lugara M., Fulco G., Palco V., De Capua C. Monitoring of Thermal Dispersion in Indoor Environments: An InfraRed Scanner Technique. Proceedings of the 2022 IEEE International Workshop on Metrology for Living Environment, MetroLivEn 2022.

[4] Lahiri B.B., Bagavathiappan S., Jayakumar T., Philip J. (2012). Medical applications of infrared thermography: A review. Infrared Phys. Technol..

[5] Kylili A., Fokaides P.A., Christou P., Kalogirou S.A. (2014). Infrared thermography (IRT) applications for building diagnostics: A review. Appl. Energy.

[6] Bagavathiappan S., Lahiri B.B., Saravanan T., Philip J., Jayakumar T. (2013). Infrared thermography for condition monitoring—A review. Infrared Phys. Technol..

[7] Bates D., Smith G., Lu D., Hewitt J. (2000). Rapid thermal non-destructive testing of aircraft components. Compos. Part B Eng..

[8] Zhao A., Xie J., Zhao Y., Liu C., Zhu J., Qian G., Wang S., Hong Y. (2022). Fatigue limit evaluation via infrared thermography for a high strength steel with two strength levels. Eng. Fract. Mech..

[9] Ciattaglia G., Iadarola G., Minelli L., Pimpini F., Tridenti N., Senigagliesi L., Spinsante S., Gambi E. Analysis of vehicle vibration through automotive radar signal. Proceedings of the 2022 IEEE International Workshop on Metrology for Automotive (MetroAutomotive).

[10] Choi M.Y., Kang K.-S., Park J.-H., An B.-W., Kim K.-S. (2019). Estimation of Dynamic Stress Concentration Factor by Infrared Thermography Stress Analysis. J. Korean Soc. Precis. Eng..

[11] Khanjani M., Ezoji M. (2021). Electrical fault detection in three-phase induction motor using deep network-based features of thermograms. Measurement.

[12] Ruffa F., Capua C.D., Morello R., Liu Z. (2019). Temperature Sensing and Evaluation of Thermal Effects on Battery Packs for Automotive Applications. IEEE Sens. J..

[13] Ruffa F., Fulco G., Lugarà M., Filianoti P., de Capua C. A real-time smart charge controller to efficiency charge processes of LiFePO4 batteries. Proceedings of the 24th IMEKO TC4 International Symposium and 22nd International Workshop on ADC and DAC Modelling and Testing.

[14] Spinsante S., Iadarola G., Mazzocchi G., Romagnoli C. Temperature Rise in MV Switchgears: The Role of Loose Busbar Joints. Proceedings of the 25th IMEKO TC4 International Symposium and 23rd International Workshop on ADC and DAC Modelling and Testing.

[15] Mevissen F., Meo M. (2022). EUltrasonically stimulated thermography for crack detection of turbine blades. Infrared Phys. Technol..

[16] Quattrocchi A., Alizzio D., Capponi L., Tocci T., Marsili R., Rossi G., Pasinetti S., Chiariotti P., Annessi A., Castellini P. (2022). Measurement of the structural behaviour of a 3D airless wheel prototype by means of optical non-contact techniques. ACTA IMEKO.

[17] Lehtiniemi R., Fager C.M., Hynninen A.M., Aapro T., Tiilikka P., Kyyhkynen V., Rantala J. Applications of infrared thermography in electronics research. Proceedings of the 2000 Quantitative InfraRed Thermography.

[18] Meckler P., Gerdinand F. High-speed thermography of fast dynamic processes on electronic switching devices. Proceedings of the 26th International Conference on Electrical Contacts (ICEC 2012).

[19] Meola C., Boccardi S., Carlomagno G.M. (2016). Infrared Thermography in the Evaluation of Aerospace Composite Materials: Infrared Thermography to Composites.

[20] Xavier P.V.M. (2012). Nondestructive Evaluation of Materials by Infrared Thermography.

[21] Lo Savio F., La Rosa G., Bonfanti M., Alizzio D., Rapisarda E., Pedullà E. (2020). Novel Cyclic Fatigue Testing Machine for Endodontic Files. Exp. Tech..

[22] Rosa G.L., Savio F.L., Pedullà E., Rapisarda E. (2016). Developing of a new device for static and dynamic tests of Ni-Ti instruments for root canal treatment. Procedia Struct. Integr..

[23] Rosa G.L., Savio F.L., Rapisarda E.P.E. (2017). A new torquemeter to measure the influence of heat-treatment on torsional resistance of NiTi endodontic instruments. Eng. Fail. Anal..

[24] Alizzio D., Savio F.L., Bonfanti M. Numerical and experimental analysis in endodontic rotary files under cyclic fatigue or torsional stress. Proceedings of the ICYRIME 2020: International Conference for Young Researchers in Informatics, Mathematics, and Engineering.

[25] Jia Y., Gao Y. (2012). Metallurgical characterization of m-wire nickel-titanium shape memory alloy used for endodontic rotary instruments during low cycle fatigue. J. Endod..

[26] Martín B., Zelada G., Varela P., Bahillo J.G., Magán F., Ahn S., Rodríguez C. (2003). Factors influencing the fracture of nickel-titanium rotary instruments. Int. Endod. J..

[27] Xu X., Eng M., Zheng Y., Eng D. (2006). Comparative study of torsional and bending properties for six models of nickel-titanium root canal instruments with different cross-sections. J. Endod..

[28] Pedullà E., Savio F.L., Boninelli S., Plotino G., Grande N.M., Rosa G.L., Rapisarda E. (2016). Torsional and cyclic fatigue resistance of a new nickel titanium instrument manufactured by electrical discharge machining. J. Endod..

[29] Plotino G., Grande N., Melo M., Bahia M., Testarelli L., Gambarini G. (2010). Cyclic fatigue of niti rotary instruments in a simulated apical abrupt curvature. Int. Endod. J..

[30] Tao F., Zhang M., Liu Y., Nee A.Y.C. (2018). Digital twin driven prognostics and health management for complex equipment. CIRP Ann..

[31] Errandonea I., Beltrán S., Arrizabalaga S. (2020). Digital Twin for maintenance: A literature review. Comput. Ind..

[32] Reifsnider K., Majumdar P. Multiphysics stimulated simulation digital twin methods for fleet management. Proceedings of the 54th AIAA/ASME/ASCE/AHS/ASC Structures, Structural Dynamics, and Materials Conference.

[33] Riemer D. Feeding the digital twin: Basics, models and lessons learned from building an IoT analytics toolbox (Invited Talk). Proceedings of the 2018 IEEE International Conference on Big Data (Big Data).

[34] Xu Y., Sun Y., Liu X., Zheng Y. (2019). A digital-twin-assisted fault diagnosis using deep transfer learning. IEEE Access.

[35] Schirmann M., Collette M., Gose J. (2019). Ship motion and fatigue damage estimation via a digital twin. Life Cycle Analysis and Assessment in Civil Engineering: Towards an Integrated Vision.

[36] Zaccaria V., Stenfelt M., Aslanidou I., Kyprianidis K.G. (2018). Fleet monitoring and diagnostics framework based on digital twin of aero-engines. Proc. ASME Turbo Expo.

[37] Luo W., Hu T., Zhang C., Wei Y. (2019). Digital twin for CNC machine tool: Modelling and using strategy. J. Ambient Intell. Humaniz. Comput..

[38] Stojanovic V., Trapp M., Richter R., Hagedorn B., Döllner J. (2018). Towards the generation of digital twins for facility management based on 3D point clouds. Management.

[39] Mars W.V., Suter J.D., Bauman M. (2018). Computing Remaining Fatigue Life under Incrementally Updated Loading Histories.

[40] Olivotti D., Dreyer S., Lebek B., Breitner M.H. (2019). Creating the foundation for digital twins in the manufacturing industry: An integrated installed base management system. Inf. Syst. e-Bus. Manag..

[41] He R., Chen G., Dong C., Sun S., Shen X. (2019). Data-Driven Digital Twin Technology for Optimized Control in Process Systems. ISA Trans..

[42] Hu F., Wang W., Zhou J. (2023). Petri nets-based digital twin drives dual-arm cooperative manipulation. Comput. Ind..

[43] Dos Santos C.H., Barra Montevechi J.A., De Queiroz J.A., De Carvalho Miranda R., Leal F. (2021). Decision support in productive processes through DES and ABS in the Digital Twin era: A systematic literature review. Int. J. Prod. Res..

[44] Cimino C., Negri E., Fumagalli L. (2019). Review of digital twin applications in manufacturing. Comput. Ind..

[45] Grieves M., Vickers J., Kahlen F.-J., Flumerfelt S., Alves A. (2017). Digital twin: Mitigating unpredictable, undesirable emergent behavior in complex systems. Transdisciplinary Perspectives on Complex Systems: New Findings and Approaches.

[46] Tao F., Cheng J., Qi Q., Zhang M., Zhang H., Sui F. (2018). Digital twin-driven product design, manufacturing and service with big data. Int. J. Adv. Manuf. Technol..

[47] Shafto M., Conroy M., Doyle R., Glaessgen E., Kemp C., LeMoigne J., Wang L. (2010). DRAFT Modeling, Simulation, Information Technology & Processing Roadmap.

[48] Glaessgen E.H., Stargel D.S. The digital twin paradigm for future NASA and U.S. Air Force vehicle. Proceedings of the 53rd AIAA/ASME/ASCE/AHS/ASC Structures, Structural Dynamics and Materials Conference.

[49] Dhar S., Tarafdar P., Bose I. (2022). Understanding the evolution of an emerging technological paradigm and its impact: The case of Digital Twin. Technol. Forecast. Soc. Chang..

[50] Angjeliu G., Coronelli D., Cardani G. (2020). Development of the simulation model for Digital Twin applications in historical masonry buildings: The integration between numerical and experimental reality. Comput. Struct..

[51] Segovia M., Garcia-Alfaro J. (2022). Design, Modeling and Implementation of Digital Twins. Sensors.

[52] Kritzinger W., Karner M., Traar G., Henjes J., Sihn W. (2018). Digital Twin in manu-facturing: A categorical literature review and classification. IFAC-PapersOnline.

[53] Ulysses J.N., Conci A. Measuring Similarity in Medical Registration. Proceedings of the IWSSIP 17th International Conference on Systems, Signals and Image Processing.

[54] Gu Q., Zhou J. A novel similarity measure under Riemannian metric for stereo matching. Proceedings of the 2008 IEEE International Conference on Acoustics, Speech and Signal Processing.

[55] Giachetti A. (2000). Matching techniques to compute image motion. Image Vis. Comput..

[56] Perri S., Frustaci F., Spagnolo F., Spagnolo F., Corsonello P. (2020). Stereo vision architecture for heterogeneous systems-on-chip. J. Real-Time Image Proc..

